# Narcissistic Traits and Explicit Self-Esteem: The Moderating Role of Implicit Self-View

**DOI:** 10.3389/fpsyg.2016.01815

**Published:** 2016-11-22

**Authors:** Rossella Di Pierro, Simone Mattavelli, Marcello Gallucci

**Affiliations:** Department of Psychology, University of Milano-BicoccaMilano, Italy

**Keywords:** narcissism, narcissistic grandiosity, narcissistic vulnerability, explicit self-esteem, implicit self-esteem

## Abstract

**Objective:** Whilst the relationship between narcissism and self-esteem has been studied for a long time, findings are still controversial. The majority of studies investigated narcissistic grandiosity (NG), neglecting the existence of vulnerable manifestations of narcissism. Moreover, recent studies have shown that grandiosity traits are not always associated with inflated explicit self-esteem. The aim of the present study is to investigate the relationship between narcissistic traits and explicit self-esteem, distinguishing between grandiosity and vulnerability. Moreover, we consider the role of implicit self-esteem in qualifying these associations.

**Method:** Narcissistic traits, explicit and implicit self-esteem measures were assessed among 120 university students (55.8% women, *M*_age_ = 22.55, *SD* = 3.03).

**Results:** Results showed different patterns of association between narcissistic traits and explicit self-esteem, depending on phenotypic manifestations of narcissism. Narcissistic vulnerability (NV) was linked to low explicit self-evaluations regardless of one’s levels of implicit self-esteem. On the other hand, the link between NG and explicit self-esteem was qualified by levels of implicit self-views, such that grandiosity was significantly associated with inflated explicit self-evaluations only at either high or medium levels of implicit self-views.

**Discussion:** These findings showed that the relationship between narcissistic traits and explicit self-esteem is not univocal, highlighting the importance of distinguishing between NG and NV. Finally, the study suggested that both researchers and clinicians should consider the relevant role of implicit self-views in conditioning self-esteem levels reported explicitly by individuals with grandiose narcissistic traits.

## Introduction

Narcissism has been described as an extreme form of high, inflated or defensive self-esteem for a long time, often leading to confusion and overlap between the two constructs (Brummelman et al., 2016). Although the distinction between narcissism and self-esteem is becoming increasingly clear, the nature of their relationship remains a relevant empirical topic. As reported in the DSM-5 Alternative Model for Personality Disorders (American Psychiatric Association, 2013), variable and vulnerable self-esteem is one of the typical features of narcissistic pathology. Kernis (2003) suggested that narcissism is associated with fragile self-esteem, and that this fragility might be due to self-esteem instability, contingent self-esteem or discrepancy between explicit and implicit self-esteem. In fact, many studies have shown that implicit and explicit self-esteem are often uncorrelated or only weakly correlated (Hofmann et al., 2005; Bosson et al., 2008; Krizan and Suls, 2008). This discrepancy lies in the fact that implicit and explicit self-esteem assess two distinct underlying processes. In general, self-esteem can be defined as *“the evaluative aspect of self-knowledge that reflects how much people like themselves”* (Zeigler-Hill and Jordan, 2010, p. 392), and such evaluation involves both explicit and implicit processes. Explicit self-esteem results from reflective and deliberative evaluation of the self. Implicit self-esteem is a function of automatic processes (Jordan et al., 2007) and it can be defined as evaluations *“that are activated in response to self-relevant stimuli, but which are not necessarily endorsed as valid reflections of how one feels about oneself*” (Zeigler-Hill and Jordan, 2010, p. 394).

The discrepancy hypothesis has attracted the scientific interest for a long time. In particular, empirical research on the relationship between narcissism and self-esteem has been largely dominated by the “mask model,” which postulates that narcissists’ positive self-views mask deep-seated feelings of inferiority and inadequacy (Kernberg, 1975; Kohut and Wolf, 1986; Emmons, 1987). According to this model, people with high narcissistic traits should reveal very high levels of explicit self-esteem combined with relatively low levels of implicit self-esteem. Despite the predominance of the mask model, the empirical investigation of the relationship between narcissism, explicit self-esteem and implicit self-esteem has led to contrasting findings. Many of the early studies converged into the idea of a positive association between explicit self-esteem and narcissism (Jordan et al., 2003; Brown and Zeigler-Hill, 2004; Zeigler-Hill, 2006; Cain et al., 2008; Rosenthal and Hooley, 2010; Brummelman et al., 2016), as well as self-enhancement tendencies (Bosson et al., 2003), which are hallmarks of narcissism. Moreover, in line with the mask model, some studies showed that the combination of high explicit and low implicit self-esteem predicted narcissistic traits (Jordan et al., 2003; Zeigler-Hill, 2006; Boldero, 2007 Unpublished). However, a meta-analysis on these studies (Bosson et al., 2008) has provided no empirical support for such results. Also, more recent studies have failed to replicate previous results, showing non-significant interaction between implicit and explicit self-esteem in predicting narcissistic traits among non-clinical samples (Campbell et al., 2007; Gregg and Sedikides, 2010), or even an opposite pattern of association in NPD participants (Vater et al., 2013).

As stated by Bosson and Prewitt-Freilino (2007), the absence of a general consensus on the definition of narcissism and its measurement might be responsible for inconsistent findings presented above. In general, empirical research has suffered from the lack of a clear definition of narcissism for a long time, which has led to confuse adaptive narcissism with pathological narcissism. Only recently, it is developing a consensus among researchers in considering adaptive and pathological narcissism as two distinct personality dimensions (see Miller and Campbell, 2008; Pincus and Lukowitsky, 2010, for extended discussions). In general, narcissism refers to “*one’s capacity to maintain a relatively positive self-image through a variety of self-, affect-, and field-regulatory processes*” (Pincus et al., 2009, p. 365). On the one hand, adaptive narcissism reflects an emotionally resilient, extraverted form of narcissism. Individuals with adaptive narcissistic traits are able to maintain self-cohesion by eliciting confirming responses from the environment, and they are able to access to inner resources when faced with disappointments. On the other hand, pathological narcissism, which may be expressed through grandiose and vulnerable manifestations, involves impaired regulatory capacities and intense needs for validation and admiration that energize the person to seek out self-enhancement experiences (Pincus and Lukowitsky, 2010; Roche et al., 2013; Pincus et al., 2014). Specifically, narcissistic grandiosity (NG) refers to the engagement in regulatory fantasies of unlimited power, superiority and perfection, entitled attitudes and disregards for needs and feelings of others. Conversely, narcissistic vulnerability (NV) includes the conscious experience of helplessness, emptiness, envy, shame, rage, and avoidance of interpersonal relationships due to hypersensitivity to rejection and criticism (Røvik, 2001; Akhtar, 2003; Dickinson and Pincus, 2003; Ronningstam, 2005; Pincus et al., 2014).

Most of the previous studies on the relationship between narcissism and self-esteem have administered the Narcissistic Personality Inventory (NPI, Raskin and Hall, 1979), which measures predominantly adaptive features of grandiose narcissism (Watson et al., 2005–2006; Cain et al., 2008; Rosenthal and Hooley, 2010). Indeed, the NPI has showed positive associations with indicators of psychological health and personal well-being (Sedikides et al., 2004; Brown et al., 2009), and negative associations with trait neuroticism, shame, and depression (Watson et al., 1992; Rhodewalt and Morf, 1995; Sedikides et al., 2004; Miller and Campbell, 2008; Samuel and Widiger, 2008; Pincus et al., 2009). Additionally, some authors argued that the NPI partially overlaps with self-esteem measures (Brown and Zeigler-Hill, 2004; Rosenthal and Hooley, 2010). In a sense, this might explain positive associations between narcissism and explicit self-esteem found by previous studies. The recent and more refined definition of pathological narcissism has led to the development of new measures of narcissism. Among these, the Pathological Narcissism Inventory (PNI; Pincus et al., 2009). The PNI assesses maladaptive features of both NG and NV, showing good psychometric properties (Wright et al., 2010). The PNI has been used both in clinical and non-clinical samples. As stated by Roche et al. (2013), *“an individual might have a constellation of normal and pathological regulatory mechanisms, employed at different times to cope with disappointments and threats to self-concept”* (p. 237). Many studies showed that NG and NV moderately correlate (Wright et al., 2010; Fossati et al., 2014; Jaksic et al., 2014; Krusemark et al., 2015). Therefore, some authors raised doubts about the possibility to distinguish clearly between them (Miller et al., 2014). Despite this, several studies found that such scales show different patterns of external correlates (Fossati et al., 2014; Jaksic et al., 2014; Krusemark et al., 2015). In line with these findings, NG and NV showed different patterns of association with self-esteem in non-clinical samples (Pincus et al., 2009; Maxwell et al., 2011; Roche et al., 2013). All the studies univocally showed that NV predicts low levels of explicit self-esteem. Conversely, the relation between NG and self-esteem is less clear. While some studies have showed that NG has positive, though often marginal, associations with explicit self-evaluations (Trzesniewski et al., 2008; Maxwell et al., 2011; Brunell and Fisher, 2014; Crowe et al., 2016), others have found no associations (Pincus et al., 2009; You et al., 2013). As a whole, these studies highlighted that the two narcissistic manifestations show different patterns of association with explicit self-esteem and that individuals with pathological grandiosity traits do not always report inflated view of themselves.

These recent findings raise serious questions about the credibility of the description of narcissism as characterized by inflated self-esteem. Moreover, recent findings lead to a question: *Why some individuals high in grandiosity traits report inflated self-images, while others do not?.* Some authors have hypothesized that external conditions, such as exposure to others, may influence explicit self-presentations in individuals with high grandiose narcissistic traits. However, Brunell and Fisher (2014) have recently found that being exposed to the presence of others during assessment procedures do not account for response bias in self-presentations in individuals high in grandiose narcissism. To the best of our knowledge, no studies investigated whether inner psychological features, such as deep-seated self-views, might influence self-reported presentations in individuals with grandiose narcissistic traits. Previous studies have suggested that individuals with grandiosity traits can have either positive or negative implicit self-view (Jordan et al., 2003; Zeigler-Hill, 2006; Campbell et al., 2007). Other studies have shown that narcissistic patients did not differ in levels of implicit self-esteem compared to healthy controls (Vater et al., 2013; Marissen et al., 2016). Despite empirical studies not demonstrating that implicit self-esteem is significantly associated with grandiose narcissistic traits, the role of implicit self-esteem in individuals with grandiosity traits remains a relevant topic. Indeed, McGregor et al. (2007) Unpublished showed differences in grandiose narcissists’ interaction with others depended on one’s level of implicit self-esteem, suggesting that implicit self-views might determine differences in affective and behavioral expressions of grandiose narcissists.

Based on these findings, we investigated whether deep-seated self-views may determine the way individuals high in narcissistic traits (especially grandiose traits) report their self-images explicitly. Contrary to previous studies on the mask model, which investigated whether the combination of low implicit and high explicit self-esteem predicted narcissism, we focused on whether and how the combination of implicit self-views and high narcissistic traits might influence explicit self-evaluations reported explicitly by individuals. According to recent findings, we hypothesized that NV and grandiosity would have different patterns of association with explicit self-esteem. Specifically, we hypothesized that vulnerable narcissism would be associated with low explicit self-esteem (Trzesniewski et al., 2008; Maxwell et al., 2011; You et al., 2013; Brunell and Fisher, 2014), regardless of the levels of implicit self-views. Conversely, we hypothesized that implicit self-views would condition explicit self-evaluations in individuals with grandiose narcissistic traits, helping to explain contrasting findings on the relationship between NG and explicit self-esteem (Trzesniewski et al., 2008; Pincus et al., 2009; Maxwell et al., 2011; You et al., 2013; Brunell and Fisher, 2014; Crowe et al., 2016).

## Materials and Methods

### Participants

One hundred and twenty psychology students (67 women, 53 men) at the University of Milano Bicocca voluntarily participated in the study. Due to high errors rate (>25%) in the Self-esteem Implicit Association Test (Self-Esteem IAT; Greenwald and Farnham, 2000) one participant was excluded from the analyses.

The final sample of participants was composed by 119 students (67 women and 52 men) with an overall mean age of 22.55 (*SD* = 3.03, range: 18–40).

Among the sample, the majority of participants (*N* = 114) were single, 3.4% (*N* = 4) were married and only one participant was divorced. Finally, the majority of participants (*N* = 77) were unemployed, whereas 35.3% of participants (*N* = 42) were employed.

### Measures

#### Implicit Self Esteem

The Self Esteem Implicit Association Test (Self-Esteem IAT; Greenwald and Farnham, 2000) is a computerized categorization task. It measures the strength of the association between self-relevant (e.g., me, my, mine) and nonself-relevant (e.g., them, they, their) stimuli with either negative or positive words. Two lists of five personal adjectives and pronouns were used as self- or other-relevant words. Five negative and five positive words served as attribute stimuli. The Self-Esteem IAT was implemented following a five-block procedure: Blocks 1, 2, and 4 were for practice, while blocks 3 and 5 were the critical ones. In block 3, the same key (e.g., “Yellow”) was required to categorize stimuli belonging to the self and positive words, while another key (e.g., “Blue”) served as a response for either nonself-related stimuli or negative words. In block 5 the association between self-related stimuli and valenced words was reversed. An IAT score was calculated following Greenwald et al. (2003) D600 score computation algorithm and reflected the ease with which participants associated either pleasant or unpleasant words to the self-concept, such that greater scores indicated higher implicit self-esteem. The order of administration of the two critical blocks has been found to affect IAT scores such that the IAT effect is consistently larger when the blocks of congruent trials are presented before the blocks of incongruent trials. Typically, between-subjects counter-balancing is used to compensate for this order effect at the group level when the magnitude of the IAT effect is the primary consideration (see Nosek et al., 2007). However, this method does not compensate for inter-individual differences, which are the focus of the present study. For this reason all participants in the present study completed the two IAT blocks in the same order of administration (i.e., congruent block first and then incongruent block). This was done in order to control the order effect at the level of the individual rather than randomly distorting it through between-subjects counter-balancing (see Zeigler-Hill, 2006 for a similar argument). In line with previous studies on Italian non-clinical participants (e.g., Richetin et al., 2012), the internal consistency of the Self-Esteem IAT was satisfactory (α = 0.85).

#### Explicit Self Esteem

The Rosenberg’s Self-Esteem scale (RSES; Rosenberg, 1965) is a well-validated measure of global self-esteem. This scale assesses the extent to which participants believe they possess good qualities, accept their own characteristics, and have achieved personal success or experienced failure. Participants completed the 10-items with four-point scales (e.g., “I feel that I have a number of good qualities”) from 1 (strongly disagree) to 4 (strongly agree). The Italian version of the RSES showed good psychometric properties among non-clinical participants (Prezza et al., 1997), and good internal consistency (α = 0.88) in the present sample.

#### Narcissistic Grandiosity and Vulnerability

The PNI (Pincus et al., 2009) is a 52-items self-report measure, which assesses two phenotypic manifestations of narcissism (Wright et al., 2010). NG is described by dimensions of Exploitativeness (EXP), Grandiose Fantasy (GF) and Self-Sacrificing Self-Enhancement (SSSE); whereas NV/NG is described by dimensions of Contingent Self-Esteem (CSE), Hiding the Self (HS), Devaluing (DEV) and Entitlement Rage (ER). All the items use a six-point response format that ask respondents to indicate how well each statement describes themselves (from 0 = not at all like me; to 5 = very much like me). The Italian version of the PNI showed good psychometric properties both in clinical and non-clinical samples (Fossati et al., 2014). In the present study both first-order (range α: 0.67–0.93) and second-order scales (NG: α = 0.85, NV: α = 0.95) showed good internal consistency.

### Procedure

Participants were invited to participate in this study through announcements on an on-line platform for managing university research studies (Sona-Systems). A research assistant instructed participants at the beginning of the experimental session. Half of participants started the session by completing a Self-esteem IAT, followed by the Rosenberg Self Esteem scale. For the other half, the order of administration of the two measures was reversed. Then, all the participants completed the PNI (Pincus et al., 2009) and provided demographic information. The experiment was implemented using Inquisit 4.0.8.0 and took approximately 20 min. At the end of the session, participants were debriefed, thanked, and received course credits for their participation.

All materials and procedures were approved by the Ethical Committee of the University of Milano-Bicocca. All subjects gave written informed consent in accordance with the Declaration of Helsinki.

### Statistical Analyses

SPSS 21.0 (Armonk, NY, USA) was employed in all analyses (IBM Corp. Released, 2012). Multiple linear regression analyses were conducted to test whether implicit self-esteem moderated the relationship between narcissistic traits and explicit self-esteem. Specifically, this moderation model was conducted separately for NG and NV, controlling for the effect of the other one. Variables were standardized before estimating the models.

## Results

The mean, SD, and correlation of implicit self-esteem, explicit self-esteem and narcissistic traits are listed in **Table 1**. Correlational analyses showed that NV and grandiosity were positively correlated (*r* = 0.53, *p* < 0.001). As regards the first-order dimensions of the PNI, no significant correlations were found with implicit self-esteem. However, all first-order dimensions of the PNI showed negative correlations with explicit self-esteem, except for EXP which was not significantly correlated.

**Table 1:** Descriptive statistics and Correlations of the IAT, RSES, first and second-order dimensions of the PNI, and total score of the PNI.

	*M*	*SD*	1	2	3	4	5	6	7	8	9	10	11
(1) SE-IAT	0.83	0.36	_										
(2) RSES	30.13	5.30	0.05	_									
(3) PNItot	6.69	1.27	-0.06	-0.41^∗∗^	_								
(4) NG	3.55	0.64	-0.03	-0.13	0.84^∗∗^	_							
(5) NV	3.13	0.81	-0.07	-0.54^∗∗^	0.90^∗∗^	0.53^∗∗^	_						
(6) CSE	2.94	0.99	-0.08	-0.67^∗∗^	0.81^∗∗^	0.47^∗∗^	0.90^∗∗^	_					
(7) GF	3.57	1.10	-0.03	-0.29^∗∗^	0.86^∗∗^	0.87^∗∗^	0.66^∗∗^	0.60^∗∗^	_				
(8) ER	3.25	1.01	-0.09	-0.24^∗∗^	0.80^∗∗^	0.55^∗∗^	0.82^∗∗^	0.68^∗∗^	0.63^∗∗^	_			
(9) DEV	2.72	0.87	-0.81	-0.52^∗∗^	0.76^∗∗^	0.39^∗∗^	0.89^∗∗^	0.75^∗∗^	0.51^∗∗^	0.62^∗∗^	_		
(10) EXP	3.25	0.84	-0.04	0.30^∗∗^	0.23^∗^	0.56^∗∗^	-0.08	-0.16	0.19^∗^	0.05	-0.08	_	
(11) HS	3.63	0.93	0.02	-0.43^∗∗^	0.69^∗∗^	0.36^∗∗^	0.80^∗∗^	0.61^∗∗^	0.49^∗∗^	0.47^∗∗^	0.67^∗∗^	-0.08	_
(12) SSSE	3.84	0.70	0.01	-0.25^∗∗^	0.69^∗∗^	0.72^∗∗^	0.51^∗∗^	0.55^∗∗^	0.60^∗∗^	0.47^∗∗^	0.38^∗∗^	0.04	0.33^∗∗^

*N = 119; SE-IAT, Self-Esteem Implicit Association Test; RSES, Rosemberg Self-Esteem Scale; PNItot, Pathological Narcissism Inventory total score; NG, narcissistic grandiosity; NV, narcissistic vulnerability; CSE, Contingent Eelf-Esteem; GF, Grandiose Fantasy; ER, Entitlement Rage; DEV, Devaluing; EXP, Exploitativeness; HS, Hiding the Self; SSSE, Self-Sacrificing Self-Esteem.*

*^∗^p < 0.05; ^∗∗^p < 0.001.*

We did not find any significant correlation between implicit self-esteem and either vulnerability (*r* = -0.07, *p* = 0.466) or grandiosity (*r* = -0.03, *p* = 0.717). Also, the correlation between implicit self-esteem and the PNI total score was not significant (*r* = -0.06, *p* > 0.517). Conversely, explicit self-esteem negatively correlated with the PNI total score (*r* = -0.41, *p* < 0.001) and NV (*r* = -0.55, *p* < 0.001), while a non-significant correlation was found between explicit self-esteem and NG (*r* = -0.13, *p* = 0.170). Finally, participants scored on average significantly higher on NG than on NV [paired-sample *t*(118) = 6.35, *p* < 0.001, standardized mean difference = 0.42].

We estimated two multiple regression models in order to test how NV and NG predicted explicit self-esteem, considering their interaction with implicit self-esteem (**Table 2**). The first model considered NV as predictor with implicit self-esteem as moderator, controlling for NG, which was entered as covariate in the model. The model explained 58.25% of the whole variance. A significant average association between NV and explicit self-esteem was found, with non-significant effect of implicit self-esteem in qualifying this relationship. Hence, the higher NV, the lower explicit self-esteem.

**Table 2:** Multiple regression analyses: testing the moderating effect of implicit self-esteem on the association between narcissism and explicit self-esteem.

	RSES				
	β	*t*	*R^2^*	*df*	*F*
Model 1^a^			0.34^∗∗^	4	14.63
NV	-0.65^∗∗^	-7.37			
SE-IAT	0.01	0.18			
NV^∗^SE-IAT	0.07	1.06			
NG	0.22^∗^	2.53			
Model 2^b^			0.34^∗∗^	4	15.78
NG	0.26^∗∗^	2.89			
SE-IAT	-0.02	-0.22			
NG^∗^SE-IAT	0.17^∗^	2.04			
NV	-0.67^∗∗^	-7.60			

*N = 119; NV, Narcissistic Vulnerability; SE-IAT, Self-Esteem Implicit Association Test; NG, Narcissistic Grandiosity; RSES, Rosemberg Self-Esteem Scale.*

*^a^Model 1: association between NV and RSES considering the moderation effect of SE-IAT and controlling for the effect of NG; ^b^Model 2: association between NG and RSES considering the moderation effect of SE-IAT and controlling for the effect of NV. ^∗^p < 0.05; ^∗∗^p < 0.001.*

**FIGURE 1:**
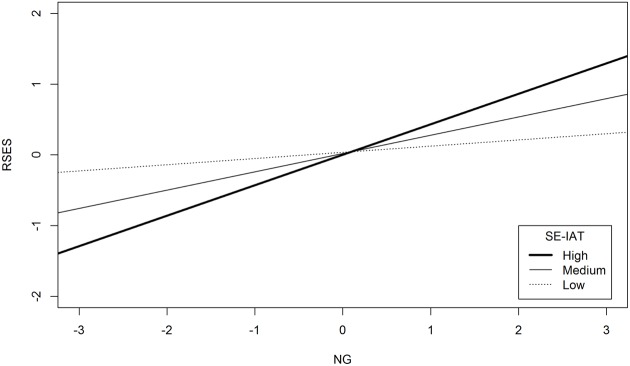
**The moderating role of implicit self-esteem on the relationship between narcissistic grandiosity and explicit self-esteem.** *N* = 119; RSES, Rosemberg Self-Esteem Scale; NG, Narcissistic Grandiosity; SE-IAT, Self-Esteem Implicit Association Test. High = 1 SD above the mean of SE-IAT; Medium = mean of SE-IAT; Low = 1 SD below the mean of SE-IAT.

With the same approach, we tested whether NG predicted explicit self-esteem, considering implicit self-esteem as a potential moderator of this relationship and controlling for NV. The whole model explained 59.70% of variance. Results showed a positive association between NG and explicit self-esteem, and this relationship was significantly moderated by implicit self-esteem. As showed in **Figure 1**, at either medium or high levels of implicit self-esteem, *B* = 0.26, *t*(114) = 2.89, *p* = 0.005 and *B* = 0.43, *t*(114) = 3.19, *p* = 0.002 respectively, the higher NG the higher explicit self-esteem. Conversely, when implicit self-esteem was low, NG and explicit self-esteem were no longer related, *B* = 0.09, *t*(114) = 0.82, *p* = 0.412.

## Discussion

The present study investigated the relationship between the two phenotypic manifestations of narcissism and explicit self-esteem. We started from the assumption that inconsistencies in previous empirical findings could be due to limitations inherent to the definition of narcissism and its assessment measures (Bosson et al., 2008; Cain et al., 2008). Recent findings on pathological narcissistic traits have showed univocally that NV is linked to low levels of explicit self-esteem, while the association between NG and explicit self-esteem is still uncertain. In the present study, we investigated whether vulnerable and grandiose narcissism have different patterns of associations with explicit self-esteem. Moreover, we tested the impact of deep-seated self-views in determining the way individuals high in narcissistic traits report explicit self-evaluations.

Our results confirmed previous studies showing moderate correlations between NG and NV scores among non-clinical samples (range: 0.50-0.66; Thomas et al., 2012; You et al., 2013; Fossati et al., 2014; Jaksic et al., 2014). The moderate correlation between the two narcissistic dimensions has led some authors to doubt about the possibility to measure separately NG and NV (Miller et al., 2013). Despite this, several studies have found that the two narcissistic manifestations show different patterns of association with anxiety, empathy capabilities, personality dimensions, attachment styles and interpersonal attitudes (Thomas et al., 2012; Roche et al., 2013; Wright et al., 2013; You et al., 2013; Fossati et al., 2014). Also our study supports the idea that NG and NV relate differently with distinct levels of explicit self-esteem, therefore confirming that they represent correlated but dissociable features of narcissism (Pincus, 2013; Pincus et al., 2014).

As in recent studies (Pincus et al., 2009; Maxwell et al., 2011; Roche et al., 2013), NV was associated with low levels of explicitly reported self-esteem. Moreover, results showed that this association was not qualified by one’s level of implicit self-view, suggesting that the relationship between vulnerable narcissistic traits and explicit self-esteem is stable and univocal. Therefore, regardless of one’s self-evaluation at an implicit level, vulnerable narcissistic traits seem to lead to a conscious (explicit) experience of the self as worthless. This is in line with the definition provided by Pincus et al. (2014), which describes vulnerable narcissism as characterized by conscious experience of feelings of helplessness and emptiness.

Our findings are in line with recent studies which have shown that grandiose narcissistic traits are not always associated with high positive explicit self-evaluations (Trzesniewski et al., 2008; Maxwell et al., 2011; You et al., 2013; Brunell and Fisher, 2014). One possible explanation for these findings might concern the role of implicit self-views in determining such association. As recently showed, implicit self-views can account for differences in interpersonal behaviors and affects explicitly reported by individuals high in grandiose traits (McGregor et al., 2007 Unpublished). Similarly, our results have confirmed the role of implicit self-views in determining differences in explicit expressions of self-esteem in individuals with high grandiose traits. Consistent with Millon (1981), who affirmed that inflated self-esteem of narcissists should be sustained by overblown inner representations of the self, our study showed that inflated self-evaluations were reported explicitly by individuals high in grandiose narcissism only when they had positive implicit self-views. On the contrary, when individuals high in grandiose narcissistic traits did not have implicit positive self-views, they did not report inflated explicit self-esteem. This suggests that, only at positive levels of implicit self-view, grandiose narcissistic traits promote the exaggeration of one’s value and attributes at an explicit level; while at negative levels of implicit self-view, no explicit exaggeration tendencies are associated with grandiose narcissistic traits. Overall, our results suggest the existence of a difference in the way grandiose narcissists express explicitly their self-esteem, and this difference may reflect the difference between covert and overt expressions of grandiose narcissistic traits (Pincus et al., 2009). As described by Pincus et al. (2014), NG reflects the tendency to seek out self-enhancement experiences through attitudes of grandiosity and superiority. Such grandiosity may be expressed either overtly, through exhibitionistic behaviors, or covertly, providing emotional or instrumental support to others and concurrently experiencing the situation as the evidence of one’s own specialness. Therefore, covert expressions of grandiosity do not involve explicit self-aggrandizement attitudes, but more often attitudes of helpfulness and willingness. Considering our results in the light of the distinction between covert and overt expressions, grandiose narcissists with high implicit self-esteem could be more likely to express their narcissistic traits through overt attitudes, such as aggrandizing their explicit self-presentations. On the contrary, grandiose narcissistic individuals with low implicit self-esteem could be more likely to show their grandiosity through covert expressions, and therefore not describing themselves through inflated self-views. However, given the lack of previous findings on the role of implicit self-views in determining explicit self-evaluations in grandiose narcissists, further research is needed to test this potential explanation.

Finally, although not central for the present study, it is interesting to notice that at low levels of narcissistic traits, individuals with positive implicit self-view reported lower levels of explicit self-esteem than individuals with less positive implicit self-view. Recent studies on modesty offer a potential explanation for this interesting finding. Modesty can be defined as the public under-representation of one’s positive traits and abilities (Cialdini et al., 1998). Some recent studies showed that modesty is often associated with discrepancy in self-evaluation, in the direction of high implicit but low explicit self-evaluations (Schröder-Abé et al., 2007; Gerstenberg et al., 2014). As people with grandiose narcissistic tendencies are highly motivated to exhibit a positive image of themselves in order to receive attention and admiration, people with low grandiose narcissistic tendencies may be less motivated to present a self-image that is positive as the self-image they have internally. Therefore, for people with low grandiose narcissistic tendencies it is plausible that the higher their implicit self-esteem the more they under-represent their positive attributes. Further studies should investigate this hypothesis and test the role of modesty traits in conditioning self-esteem levels (both explicit and implicit) among people with low grandiose narcissistic traits.

The results of the current study can be better understood in the context of the study’s limitations. Unlike previous studies (Jordan et al., 2003; Zeigler-Hill, 2006; Boldero, 2007 unpublished), we proposed a change of perspective in the analysis of the relationship between narcissism and self-esteem. As stated by Brummelman et al. (2016), narcissism and self-esteem have been considered as overlapping constructs for a long time. Previous studies often started from this assumption when examining the association between narcissism and self-esteem. Recently, the distinction between the two constructs has been increasingly clear, and some studies have showed that narcissism is not always associated with positive self-view (Pincus et al., 2009; Maxwell et al., 2011; Roche et al., 2013). After all, whether narcissistic traits might be indicative of one’s levels of explicit self-esteem, it seems less plausible that one’s level of explicit self-esteem might be indicative of narcissistic traits. We hypothesized that stable narcissistic traits can predict the way people report explicit self-evaluations, rather than the opposite pattern. However, in interpreting the present findings we acknowledge that cross-sectional design allows correlational rather than causal relationships to be established. Therefore, further studies should better investigate this hypothesis. Moreover, we acknowledge that the measure we used to assess implicit self-esteem activates respondents’ communal self-view more than their agentic one (Campbell et al., 2007). Since this may influence the study of the association between narcissism and implicit self-esteem, further studies are needed to replicate our findings considering both agentic and communal implicit self-views separately. Moreover, the weak associations found between implicit self-esteem measures (Bosson et al., 2008) would recommend the administration of other implicit measures of self-esteem (e.g., Name Letter Test, Nuttin, 1985). Also the chosen narcissism measure might suffer from some limitations. Although, the PNI has showed good psychometric properties across studies in several social and cultural contexts (Pincus, 2013), some authors have recently raised some critiques about the use of the PNI due to the fact that its pattern of correlations with relevant dimensions deviates from those exhibited by other grandiosity measures (Miller et al., 2014, 2016). After all, as stated by Wright (2016) results from these studies might be understandable and expected considering the construct of NG measured by the PNI. The PNI was developed with the intent to capture clinical aspects of narcissism, which were not described by the DSM-IV NPD diagnosis (American Psychiatric Association, 2000). In fact, DSM-IV NPD have showed limited utility over time because of its partial description of narcissistic pathology, which was focused only on behavioral aspects of overt grandiosity. In this sense, the PNI assesses both grandiosity and vulnerability in their overt and covert expressions. We believe that investigating the relationship between narcissistic traits and self-esteem using other self-report measures, such as the Five Factor Narcissism Inventory (FFNI; Glover et al., 2012), should be encouraged, in order to understand whether similar results might be found. Finally, the present study included university students. Although results on the relationship between narcissistic traits, explicit and implicit self-esteem are promising, the present model should be investigated in clinical samples in order to test whether results might be replicated.

## Conclusion

Findings from the present study showed that the relationship between narcissism and self-esteem is not univocal, supporting the importance of distinguishing between the two phenotypic manifestations of narcissism as well as the need to include implicit self-esteem measures. In fact, while NV showed univocal association with low levels of explicit self-esteem, the way individuals with high traits of NG reported explicit self-esteem was conditioned by their implicit self-view. In this sense, the study suggests that clinicians should consider explicit self-evaluations but also implicit self-views when treating individuals who show grandiose narcissistic traits.

## Author Contributions

RDP contributed to prepare the study design, to organize the recruitment of the sample, to analyze the data, and to write all sections of the manuscript. SM contributed to prepare the study design, to collect and analyze the data, and to write the methods section of the manuscript. MG contributed to prepare the study design and to supervise the research team. All the authors reviewed and approved manuscript for publication.

## Conflict of Interest Statement

The authors declare that the research was conducted in the absence of any commercial or financial relationships that could be construed as a potential conflict of interest.
